# Outcomes of two randomized controlled trials, employing participants recruited through Mechanical Turk, of Internet interventions targeting unhealthy alcohol use

**DOI:** 10.1186/s12874-019-0770-4

**Published:** 2019-06-14

**Authors:** John A. Cunningham, Alexandra Godinho, Nicolas Bertholet

**Affiliations:** 1 Centre for Addiction and Mental Health, 33 Russell St., Toronto, Ontario M5S 2S1 Canada; 2 Department of Psychiatry, University of Toronto, Toronto, M5T 1R8 Canada; 3 Dalla Lana School of Public Health, University of Toronto, Toronto, M5S 1A1 Canada; 4 Alcohol Treatment Center, Department of Community Medicine and Health, Lausanne University Hospital, Lausanne, Vaud Switzerland

**Keywords:** Amazon mechanical Turk, MTurk, Internet intervention, Online web, Data collection, Research methods, Alcohol

## Abstract

**Background:**

Two randomized controlled trials (RCTs) were conducted to explore the utility of the Mechanical Turk (MTurk) crowdsourcing platform to conduct rapid trials evaluating online interventions for unhealthy alcohol use.

**Methods:**

Both trials employed a staged recruitment procedure where participants who drank in an unhealthy fashion were identified using a baseline survey and then invited to take part in a 6-month follow-up. Participants in both trials were randomized to receive one of several different online interventions or to a no intervention control condition. In study 1, the online interventions were password protected and only those who accessed the study portal were randomized to condition. In study 2, participants were directed to free-of charge interventions and asked to send a screenshot of the intervention to demonstrate that they had complied.

**Results:**

Participants reporting unhealthy alcohol use were recruited fairly rapidly. Large numbers of screeners were completed (Study 1: *n* = 4910; Study 2: *n* = 5812), found eligible (Study 1: *n* = 3741; Study 2: *n* = 4095), and randomized to condition (Study 1: *n* = 511; Study 2: *n* = 878). Fair follow-up rates were observed at 6 months for each study (Study 1: 82%; Study 2: 66%). Neither trial was able to clearly demonstrate that providing access to the online interventions lead to increased reductions in alcohol use as compared to the control group.

**Conclusions:**

While recruitment through a crowdsourcing platform is rapid and relatively low cost, it is possible that the lack of impact of the online websites employed in these trials could be due to the source of participants rather than the lack of efficacy of the interventions.

**Trial registration:**

ClinicalTrials.gov # NCT02977026 and NCT03060135.

## Background

Over the last two decades, there has been a significant increase in the availability of Internet interventions for unhealthy alcohol use [1–3]. This work is important because unhealthy alcohol use causes significant harm to the individual and to society, and is one of the primary contributors to the modifiable burden of disease globally [4]. Further, the large majority of people who drink in an unhealthy fashion never access treatment, including the receipt of brief interventions in primary healthcare settings [5]. Fortunately, many people with unhealthy alcohol use appear interested in other options to address their alcohol consumption [6], stimulating research on alternate ways to provide access to care [3].

A challenge with evaluating Internet interventions for unhealthy alcohol use, as with interventions for many other modifiable health behaviours, is the need for the recruitment of large numbers of participants. This is because the interventions generally have fairly small effect sizes, thus requiring a large sample to have enough power to test for an impact of the intervention [1, 2]. Further, while randomized controlled trials (RCTs) are a powerful technique to provide evidence as to whether an intervention is effective, each individual trial, no matter its quality, is of limited use as a definitive indicator of whether an intervention works. Multiple trials, ideally from independent research groups, are required to build an adequate evidence base of the effectiveness of any intervention. Once such an evidence base is established, interventions also benefit from continued development to increase their impact, to make them more attractive to the participant, or to establish their efficacy in specialized populations (e.g., those with co-occurring disorders).

Such research is expensive and generally time consuming. It would be extremely valuable to the development of an evidence base of Internet interventions to have a readily available, high quality, and low cost source of participants for research trials. While online advertisements (e.g., on Facebook or through Google AdWords) can be successful, this success can be variable over time and the cost of advertisements can easily build up. Other areas of the social sciences have started to rely heavily on crowdsourcing platforms, such as Mechanical Turk (MTurk), for recruiting people to participate in online surveys and in experimental trials that can be conducted online [7, 8]. Much has be written on the strengths and weaknesses of MTurk as a ready source of participants, but there is general agreement that there is the potential, given the number of people registered as ‘workers’ on MTurk (upwards of 500,000), to recruit large numbers of participants for a relatively low cost (e.g., US$1 is generally regarded as good pay for completing a 10 min survey).

While there are many published manuscripts reporting on research employing MTurk workers as participants, there is only very limited research to-date on whether MTurk might also be a good source of participants for Internet intervention research [9]. Previous studies have demonstrated that it is possible to recruit participants with clinically relevant symptoms through MTurk, such as participants who report drinking in an unhealthy fashion [10]. We sought to establish whether such participants might be willing to engage with Internet interventions and whether these participants can then be followed up over time. The eventual goal was to identify a source of large numbers of participants that could be employed during the development and preliminary evaluation stage of Internet interventions. To this purpose, we conducted an initial randomized controlled trial (RCT) employing MTurk workers who reported unhealthy alcohol use as participants and an online brief intervention that had demonstrated efficacy in seven previous randomized trials [11–17]. The assumption was that the intervention was an active one and that an absence of observed effect of the intervention with MTurk workers could be used as evidence that MTurk was a poor source of participants (and any evidence of impact of the Internet intervention among MTurk worker participants would indicate that this might be a good source of participants for future trials). Briefly, our initial study found that we could quickly register relevant participants for a trial (425 in 3 h), that follow-up rates at 3 months were good (85%), and that there was some limited evidence of impact of the Internet intervention on levels of alcohol consumption among participants [17]. There were also some indications of the limitations of MTurk as a source of participants, as the majority (62%) of participants asked to access the intervention did not do so. Given the promise and limitations of the initial trial, we sought to systematically replicate the Internet intervention trial using MTurk participants. Each of the two additional RCTs incorporated methods to encourage participants to engage with the intervention materials. The experience with recruiting the large number of participants for these trials, and others, is reported elsewhere [9]. The current manuscript reports on the outcome of two of these RCTs and the lessons learned from this experience.

## Study 1 introduction

In the first trial, we sought to compare the efficacy of a brief online personalized feedback intervention (Check Your Drinking; CYD) [12] to a more extended online intervention containing cognitive behavioural and relapse prevention techniques (Alcohol Help Centre; AHC) [18, 19]. A no intervention control condition was also included in the trial (participants were asked what components of the CYD they thought would be helpful after being provided with a brief description of each of the components). This style of control condition was chosen, rather than a waiting list control, because of evidence that waiting list controls can artificially increase the difference observed between experimental conditions [20].

## Study 1 methods

The first two steps of the recruitment procedure were the same in studies 1 and 2. In step 1, an advertisement was placed on the MTurk portal asking for participants to complete a survey on people’s drinking and stating that only current drinkers were asked to participate. The advertisement also stated that the survey would take less than 15 min to complete and that the reimbursement was US$1.50. Participants who clicked on the link were provided with a brief description of the study and asked to complete a brief eligibility survey (18 years or older and drink weekly or more often). The potential participant’s MTurk worker number was captured along with the eligibility screener so that each potential participant could only complete the eligibility survey once for any of the online alcohol intervention trials we conducted (i.e., MTurk workers were excluded from checking for eligibility in future trials conducted by our research group after they had completed one eligibility screener). Participants who met eligibility criteria were then sent to an online consent form (step 2 of recruitment) which included the information that the survey was about their alcohol use and, further, that some people would be asked if they were interested in participating in another study (but indicated that we did not know if they would be asked). Those consenting to participate then completed the baseline survey. In addition to demographic characteristics, participants were asked about their alcohol consumption using validated questionnaires that have been used in online studies, including amount of drinking in a typical week, highest number of drinks on one occasion, the Alcohol Use Disorders Identification Test (AUDIT) [21], and a modified version of the Wechsler et al. [22], items assessing alcohol consequences (an additional item was added regarding driving while under the influence of alcohol). The survey also contained attention check questions.

Participants who completed the survey were considered for step 3 of the recruitment process (i.e., invitation to take part in the RCT) if they answered all attention check questions correctly, stated that they had provided accurate data, and that we should keep the data (the latter two questions were accompanied by the statement that their responses would not affect payment). Further, only people who reported drinking at an unhealthy level - defined as an AUDIT score of 8 or more and typical weekly alcohol consumption of at least 15 drinks per week - were invited to participate in the RCT. A drink was defined as 12 oz. of beer, 5 oz. of wine, 3 oz. of fortified wine, or 1.5 oz. of liquor. A weekly drinking criterion was added to the AUDIT score of 8 or more criterion employed in the initial trial because there were many participants in the initial study who, while scoring 8 or more on the AUDIT, were nevertheless reporting alcohol consumption below low risk drinking guidelines (for the logic behind using the same drinking cut-off for males and females, please see [23]). Those participants found eligible were presented with an online page after the completion of the survey which informed them that, in addition to the payment for the initial survey, they could also participate (if interested) in another survey in 6 months’ time asking about their drinking over the past 6 months. Further, they were informed that some participants would be asked to look at and give their impressions of materials that had been designed to help people with their alcohol consumption. Participants were informed that they would be paid $10 for completing the follow-up survey. Participants indicated their agreement to take part in the 6-month follow-up survey by clicking on the link provided. Those participants who were not eligible for the 6-month follow-up (and the RCT) were thanked for their participation in the baseline survey. All participants were then paid for completing the recruitment survey.

Participants agreeing to take part in study 1 were thanked for agreeing to take part in the study and told that we would send them information regarding next steps using their MTurk worker email account. The email contained information on how to log into the study portal along with a unique access code for each participant. Participants were informed that only participants who logged onto the study portal would receive a 6-month follow-up survey and be eligible for the $10 compensation. Logging onto the study portal (separate website from MTurk) with the unique code randomized participants into one of the three study conditions - provided access to the CYD, the AHC, or the control condition questionnaire.

### Outcome variables and analysis plan

The primary outcome variable for studies 1 and 2 was the number of drinks consumed in a typical week. In addition, for study 1, the three item consumption sub scale from the AUDIT (the AUDIT-C) was also defined as a primary outcome variable (the AUDIT-C was a secondary outcome variable in study 2) [24]. Other secondary outcome variables were largest number of drinks on one occasion and the number of consequences experienced. Prior to the analyses of the primary and secondary outcomes, outliers found to be more than 3.29 standard deviations above the sample mean were removed, and variables with a non-normal distribution were log-transformed. The pre-specified analysis plan involved generalized liner modelling employing an intent-to-treat approach with missing data handled using a maximum likelihood approach.

Ethics approval for both studies 1 and 2 was provided by the standing research ethics board of the Centre for Addiction and Mental Health.

## Study 1 results

**Fig. 1:**
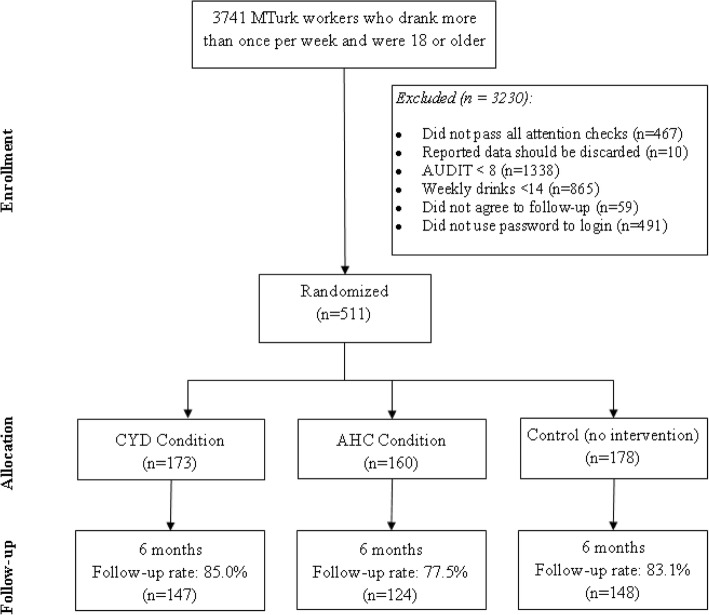
Trial consort diagram for Study 1

Recruitment occurred during December 2016. In that time, a total of 3741 participants were found eligible to complete the baseline survey (i.e. drank more than once per week and were 18 years or older), of which 1002 were found eligible and agreed to participate in the follow-up survey. Overall, 511 potential participants used their assigned password to login to the study portal and were randomized to conditions (173 in the CYD condition; 160 in the AHC condition; 178 in the no intervention control condition). Follow-up occurred in June 2017. A total of 421 (82.4%) participants completed the 6 month follow-up; however the follow-up data of 2 participants were excluded from analyses due to inaccurate data reporting (i.e. when asked, they stated that they had not provided accurate follow-up data and that their data should be deleted). See Fig. 1 for a consort diagram.

**Fig. 2:**
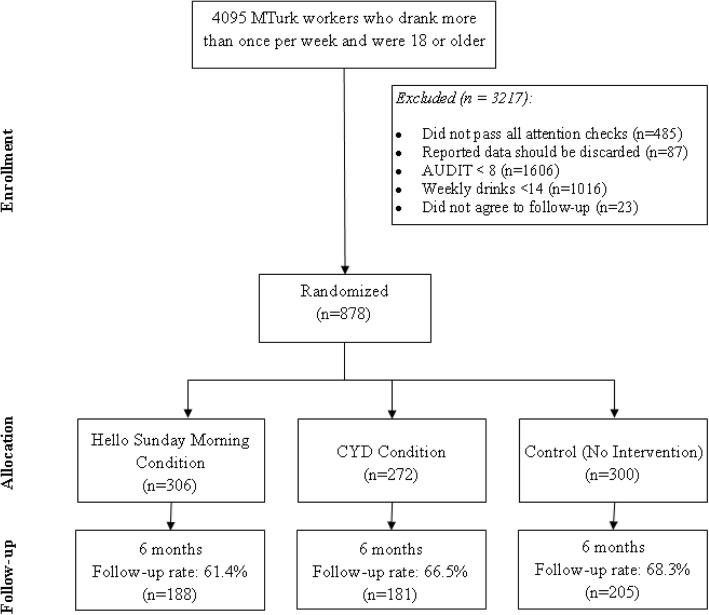
Trial consort diagram for Study 2

The average age (SD) of the sample was 36.3 (10.2), 56% were male, the majority were white (84%), nearly half (48%) were married or living in a common law relationship, 63% had some post-secondary education, 70% were full-time employed (including full-time self-employed), and 22% reported a family income of less than US$20,000 per year. At baseline, participants had a mean (SD) AUDIT score of 17.7 (7.0), drank 27.3 (11.7) drinks in a typical week, reported 11.1 (5.2) drinks as the most they drank on one occasion in the past 6 months, and experienced 3.5 (2.3) alcohol consequences. Nonetheless, only 23% of participants said that they had ever accessed treatment in relation to their alcohol use.

Bivariate comparisons found no significant (*p* > .05) differences in baseline demographic and drinking variables across all 3 interventions, with the exception of marital status; those randomized into the no-intervention control condition were less likely to be married as compared to those randomized into the AHC intervention condition (39% vs. 54%; *p* = 0.016). Lastly, 6-month follow-up rates did not appear to significantly differ across the control, CYD or AHC condition (83% vs. 85% vs. 78% respectively, *p* = 0.184).

Separate mixed-effects models were conducted to examine the effect of time, intervention condition, and the time by intervention interaction on both primary (i.e. # of drinks per week, AUDIT-C scores) (Table 1) and secondary variables (i.e. largest number of drinks consumed on one occasion, number of alcohol consequences experienced) as specified in the analysis plan (Table 2). The models revealed that the sample as a whole experienced significant reductions on the number of drinks per week and AUDIT-C scores from baseline to 6-months, however no differences in the level of reductions was observed between each of the intervention conditions and the no-intervention control condition. Similarly, the sample as a whole also experienced significant reductions over time in reported highest number of drinks consumed on one occasion and number of experienced alcohol consequences. While the level of reduction for the highest number of drinks reported on one occasion did not vary across conditions over time (i.e. time by condition interaction was not significant), a significant time by condition interaction was observed for the number of drinking-related consequences reported over time. In particular, participants in the CYD condition experienced significantly fewer drinking-related consequences than those randomized into the no-intervention control condition (estimate = − 0.09, 95% confidence interval [CI] = − 0.15 to − 0.02, *p* = 0.011), however no significant differences were found between the AHC condition and the no-intervention condition.

**Table 1:** Mixed-effect models results of time, intervention, and time by intervention interaction on drinks in a typical week and AUDIT-C (Study 1)

Effect	Drinks in a typical week^ab^			AUDIT – C^a^		
	Estimate ± SE	*t*	*p*	Estimate ± SE	*t*	*p*
Intercept	1.41 ± 0.02	57.81	< 0.001	8.48 ± 0.17	50.08	< 0.001
Time (Ref: Baseline)	−0.30 ± 0.03	−9.14	< 0.001	−1.80 ± 0.21	−8.44	< 0.001
Condition (Ref: Control condition)						
CYD condition	− 0.02 ± 0.03	−0.57	0.569	−0.24 ± 0.24	−1.00	0.319
AHC condition	−0.02 ± 0.04	−0.55	0.583	−0.03 ± 0.25	−0.14	0.893
		***F***	***p***		***F***	***p***
Time x Intervention (Ref: Baseline x control condition)		1.85	0.158		1.10	0.333

*SE* Standard Error

^a^primary outcome variable

^b^Model was conducted using the log transformation of the outcome variable

**Table 2:** Mixed-effect models results of time, intervention, and time by intervention interaction on highest # of drinks in a day and # of consequences (Study 1)

Effect	Highest # drinks on one occasion^a^			# of alcohol consequences^a^		
	Estimate ± SE	*t*	*p*	Estimate ± SE	*t*	*p*
Intercept	1.02 ± 0.02	54.89	< 0.001	0.47 ± 0.02	21.48	< 0.001
Time (Ref: Baseline)	−0.14 ± 0.02	−6.17	< 0.001	−0.10 ± 0.02	−4.23	< 0.001
Condition (Ref: Control condition)						
CYD condition	−0.03 ± 0.03	−1.10	0.272	−0.002 ± 0.03	−0.06	0.955
AHC condition	−0.03 ± 0.03	−1.02	0.308	−0.03 ± 0.03	−1.09	0.274
		***F***	***p***		***F***	***p***
Time x Intervention (Ref: Baseline x control condition)		1.91	0.149		3.35	0.036

*SE* Standard Error

^a^Models were conducted using the log transformation of the outcome variable

## Study 2 introduction

There are a number of free-of-charge websites available offering help to people with unhealthy alcohol use. In study 2, we sought to determine whether it was possible to conduct a trial evaluating the impact of Internet intervention websites that we did not have direct control over. After examining several free-of-charge online interventions for suitability for an American audience (i.e., MTurk workers) we chose Hello Sunday Morning (HSM) [25], comparing it to Check Your Drinking (CYD; public access version) as the active control and to a no intervention control condition. The objectives of study 2 were to test whether it was possible to conduct the trial in such a way that the participants demonstrated that they had accessed the intervention, and to examine whether there was any evidence of impact of the intervention. Further, we wanted to create a design that would equalize contact and payment amount between intervention and control conditions [26].

## Study 2 methods

The initial recruitment process was identical to study 1. Those agreeing to take part in the 6-month follow-up were sent an email. Participants in the HSM and the CYD conditions received an email thanking them for participating in the study. They were asked to go to the respective public access websites (along with providing a link and instructions), complete the personalized feedback component (i.e., the full intervention for the CYD and the registration component for HSM), and to send a screenshot of the report generated by the website attached to a reply email (for which they would be paid $5). Participants in the control condition were sent an email thanking them for participating in the study and asking them to reply to the email to confirm that we were able to contact them. As with the participants in the intervention conditions, these participants were told that they would be paid $5 for completing this task. It is important to note that, unlike study 1, completing the personalized feedback component and sending a screenshot to the study team (or replying to the email for those randomized into the control condition) was not a requirement for enrolment and randomization into the study. This is the most likely explanation for the higher attrition rates detailed in the results section below.

## Study 2 results

Recruitment occurred during February and March 2017. During that time, a total of 4095 participants were found eligible to complete the baseline survey (drank more than once per week and were 18 years or older), of which 878 were found eligible and agreed to participate in the follow-up survey. These participants were randomized to conditions (306 in the HSM condition; 272 in the CYD condition; 300 in the no intervention control condition). Follow-up occurred during August and September 2017. A total of 580 (66.2%) participants completed the 6 month follow-up; however the follow-up data of 6 participants were excluded from further analyses due inaccurate data reporting. In addition, 64% (*n* = 565) of all participants replied to the initial study email (49% of those in the HSM condition; 58% of those in CYD, and 84% of those in the no-intervention control condition). See Fig. 2 for a consort diagram. 

The sample characteristics were similar to those observed in Study 1. More specifically, the average age (SD) of the sample was 33.4 (10.0), 55% were male, most were white (83%), close to half (41%) were married or living in a common law relationship, 63% had some post-secondary education, 60% were full-time employed (including full-time self-employed), and 25% reported a family income of less than US$20,000 per year. Similar drinking characteristics as in study 1 were also observed with participants having a mean (SD) AUDIT score of 18.1 (6.8), drank 27.2 (12.5) drinks in a typical week, reported 11.1 (4.7) drinks as the most they drank on one occasion in the past 6 months, and experienced 3.9 (2.3) consequences. A total of 38.4% of participants said that they had ever accessed treatment in relation to their alcohol use.

Bivariate comparisons found no significant (*p* > .05) differences in baseline demographic and drinking variables across all 3 interventions. Furthermore, 6-month follow-up rates were lower than that observed in study 1 overall, however they did not appear to significantly differ across the no intervention control, CYD or HSM intervention conditions (68% vs. 67% vs. 61% respectively, *p* = 0.181).

Four separate mixed-effects models were conducted to examine the effect of time, intervention condition, and the time by intervention interaction on the primary (i.e. # of drinks per week) and secondary variables (i.e. AUDIT-C, largest number of drinks consumed in one occasion, number of consequences experienced) as specified in the analysis plan (Tables 3 and 4). The model revealed significant reductions in the number of drinks per week from baseline to 6-months but no significant differences (*p* > .05) in the level of reductions between intervention and control conditions. Similarly, the sample as a whole also experienced significant reductions over time in all of the secondary outcome variables (i.e. AUDIT-C, highest number of drinks on one occasion, and the number of consequences experienced), however none of the reductions observed over time differed across the intervention conditions.

**Table 3:** Mixed-effect models results of time, intervention, and time by intervention interaction on drinks in a typical week and AUDIT-C (Study 2)

Effect	Drinks in a typical week^ab^			AUDIT - C		
	Estimate ± SE	*t*	*p*	Estimate ± SE	*t*	*p*
Intercept	1.40 ± 0.02	76.01	< 0.001	8.42 ± 0.12	67.53	< 0.001
Time (Ref: Baseline)	−0.26 ± 0.03	−9.55	< 0.001	−1.48 ± 0.17	−8.83	< 0.001
Condition (Ref: Control condition)						
CYD condition	−0.004 ± 0.03	−0.15	0.879	−0.17 ± 0.18	−0.94	0.347
AHC condition	−0.005 ± 0.03	−0.20	0.844	−0.11 ± 0.18	−0.61	0.545
		***F***	***p***		***F***	***p***
Time x Intervention (Ref: Baseline x control condition)		1.17	0.312		0.86	0.424

*SE* Standard Error

^a^primary outcome variable

^b^Model was conducted using the log transformation of the outcome variable

**Table 4:** Mixed-effect models results of time, intervention, and time by intervention interaction on secondary outcome variables of Study 2

Effect	Highest # drinks on one occasion^a^			# of alcohol consequences^a^		
	Estimate ± SE	*t*	*p*	Estimate ± SE	*t*	*p*
Intercept	1.02 ± 0.01	78.29	< 0.001	0.50 ± 0.02	29.47	< 0.001
Time (Ref: Baseline)	−0.11 ± 0.02	−6.48	< 0.001	−0.12 ± 0.02	−6.19	< 0.001
Condition (Ref: Control condition)						
CYD condition	−0.02 ± 0.02	−1.30	0.192	−0.01 ± 0.02	−0.58	0.565
AHC condition	−0.02 ± 0.02	−1.00	0.319	− 0.01 ± 0.02	−0.48	0.634
		***F***	***p***		***F***	***p***
Time x Intervention (Ref: Baseline x control condition)		2.18	0.114		0.29	0.748

*SE* Standard Error

^a^Models were conducted using the log transformation of the outcome variable

## Study 1 and 2 conclusions

The two trials demonstrated successful methods of promoting initial access of online interventions in RCTs - the first to restrict the trial to those who accessed a password controlled study intervention portal and the second by paying participants to provide a screenshot of the intervention under study. Both methods could be successfully employed in other intervention trials, with the second being of particular relevance when the intervention under study is not under the researcher’s control. However, neither method is targeted at promoting continued use of an intervention; something that may be required in order to promote reductions in alcohol consumption. There were also some positive aspects to using Mechanical Turk as a source of participants. Recruitment was rapid (7 and 32 days respectively), relatively inexpensive, and yielded large samples of participants who reported drinking in an unhealthy fashion. However, recruitment slowed down substantially over time, underlining the fact that crowdsourcing platforms are finite. A more thorough analysis of recruiting for alcohol intervention research on Mturk (i.e. cost, time to recruit, and eligible sample sizes) has been reported elsewhere [9, 17].

Because there were no significant differences between intervention conditions in the primary outcome variables, the study found little evidence for the potential of MTurk as a possible source of participants for Internet intervention trials. While evidence for the AHC and HSM as active interventions is equivocal, the CYD has several previous trials demonstrating efficacy of the intervention. In particular, previous trials have consistently demonstrated that participants who are provided with this normative feedback program report significantly reduced alcohol consumption on comparable measures (i.e. number of drinks consumed per week) [11, 12, 14–16]. Thus, it is reasonable to assume that it is the participants, rather than the intervention, that is the cause of the lack of observable effects. Even if this assumption is valid, it still does raise the point that providing access to a known effective intervention does not necessarily guarantee an impact among all participants. This is particularly relevant with interventions such as the CYD where the intended audience is the general population of people with unhealthy alcohol use (including MTurk workers) demonstrating that a solid base of efficacy trials does not mean that the intervention will actually prove effective in every real-world setting. It is also relevant to note that study 1 was in all likelihood unable to specifically test intervention effectiveness because, while 1002 participants agreed to participate, only about half actually used their password to access the intervention portal, confirming their participation in the trial. The same holds true for study 2: although the design differed, only 58% accessed CYD.

These two trials fairly clearly demonstrate that crowdsourcing platforms are a poor source of participants for full intervention trials requiring participants to actively engage with the intervention. However, is it possible that this easy source of participants might be of use during the development stage of an intervention? The challenges are that the participants will not engage in extensive interaction with an intervention (so the intervention component under study would need to be brief), that the participants are specifically taking part for pay, and that they will most likely have completed many other surveys and studies in the past (limiting the generalizability of the results). For the studies conducted by our group, we limited the participation to only one study, but that doesn’t mean that participants did not participate in other studies focusing on drinking in the past or at the same time. There is therefore a risk of assessment reactivity. Further, any impact of the intervention will in all likelihood be short-term, as is indicated by our earlier trial demonstrating some impact of an online intervention at 3 months but that the current trials found little evidence of impact at 6 months [9]. On the other hand, MTurk workers may be less sensitive to social desirability [27], and differ by demographic (e.g. more female, younger, participants, more education, lower incomes) and traits (e.g. more anxious, less emotional stability) compared to general public samples [28, 29]. Future research should consider these differences when generalizing findings to community samples. Still, there is merit to testing whether crowdsourcing platforms are a useful source of participants for trials where small components of interventions are experimentally manipulated and outcome measures are short term and amenable to change (i.e., it may be easier to impact on ratings of intent to change or concerns about drinking rather than actual levels of alcohol consumption). As there is a clear need to develop new and effective interventions targeting unhealthy alcohol use, as well as interventions targeting other health behaviours, and because full trials are expensive in both time and resources, finding means to conduct such developmental trials (e.g., by recruiting through MTurk or other crowdsourcing platforms) would be an important contribution.

## Data Availability

Not applicable.

## Abbreviations

AUDIT: Alcohol Use Disorders Identification Test
AUDIT-C: Consumption subscale of the Alcohol Use Disorders Identification Test
MTurk: Mechanical Turk
RCT: Randomized controlled trial
